# Identification of conserved and novel miRNAs responsive to heat stress in flowering Chinese cabbage using high-throughput sequencing

**DOI:** 10.1038/s41598-019-51443-y

**Published:** 2019-10-17

**Authors:** Waqas Ahmed, Yanshi Xia, Hua Zhang, Ronghua Li, Guihua Bai, Kadambot H. M. Siddique, Peiguo Guo

**Affiliations:** 1 International Crop Research Center for Stress Resistance, College of Life Sciences, Guangzhou University, Guangzhou, 510006 China; 2 Guangzhou Academy of Agricultural Sciences, Guangzhou, 510308 China; 3 United States Department of Agriculture – Agricultural Research Service, Hard Winter Wheat Genetics Research Unit, Manhattan, Kansas 66506 United States of America; 4 The UWA Institute of Agriculture and School of Agriculture & Environment, The University of Western Australia, LB 5005, Perth, WA 6001 Australia

**Keywords:** Abiotic, Heat

## Abstract

Plant microRNAs (miRNAs) are noncoding and endogenous key regulators that play significant functions in regulating plant responses to stress, and plant growth and development. Heat stress is a critical abiotic stress that reduces the yield and quality of flowering Chinese cabbage (*Brassica campestris* L. ssp. *chinensis* var. *utilis* Tsen et Lee). However, limited information is available on whether miRNAs are involved in the regulation of heat stress in *B. campestris*. A high-throughput sequencing approach was used to identify novel and conserved heat-responsive miRNAs in four small RNA libraries of flowering Chinese cabbage using leaves collected at 0 h, 1 h, 6 h and 12 h after a 38 °C heat-stress treatment. The analysis identified 41 conserved miRNAs (belonging to 19 MIR families), of which MIR156, MIR159, MIR168, MIR171 and MIR1885 had the most abundant molecules. Prediction and evaluation of novel miRNAs using the unannotated reads resulted in 18 candidate miRNAs. Differential expression analysis showed that most of the identified miRNAs were downregulated in heat-treated groups. To better understand functional importance, bioinformatic analysis predicted 432 unique putative target miRNAs involved in cells, cell parts, catalytic activity, cellular processes and abiotic stress responses. Furthermore, the Kyoto Encyclopedia of Genes and Genomes maps of flowering Chinese cabbage identified the significant role of miRNAs in stress adaptation and stress tolerance, and in several mitogen-activated protein kinases signaling pathways including cell death. This work presents a comprehensive study of the miRNAs for understanding the regulatory mechanisms and their participation in the heat stress of flowering Chinese cabbage.

## Introduction

*Brassica* vegetable crops—including cabbage (red, green, etc.), brussels sprouts, broccoli, kale and cauliflower—belong to the Brassicaceae family, all of which are cultivated and consumed throughout the world. *Brassica* species are morphologically and phytochemically diverse. Some *Brassica* species accumulate secondary metabolites such as phenolics and glucosinolates that are beneficial to human health. Flowering Chinese cabbage (*Brassica campestris* L. ssp. *chinensis* var. *utilis* Tsen et Lee) is one of the most commonly cultivated vegetable crops in the southern part of China^1^. Demand for flowering Chinese cabbage is increasing in China as it is reasonably priced and has high nutritional value, soluble fiber and vitamin C. However, due to genetic and adverse environmental stresses, the quality and productivity of flowering Chinese cabbage have declined^2^.

Heat stress is a major environmental factor that negatively affects physiological processes, reproduction, adaptation and crop development, which in turn reduce yield^3,4^. Global climate analysis predicted that high temperature is becoming more frequent and has the potential to affect crop yields in the near future^5^. Flowering Chinese cabbage is a cryophilic vegetable crop that has been planted year-round in southern China since its domestication several hundred years ago. It often suffers from high-temperature stress in summer^6^, which not only influences plant growth and performance but results in yield reductions and even death^7^. Flowering Chinese cabbage genotypes can have obvious premature leaf senility, fine flowering stalks, significantly reduced photosynthetic capacity, and high electrolytic leakage and malondialdehyde content under high-temperature condition^8,9^.

Heat-stress tolerance in vegetable crops is a complex phenomenon involving various metabolic and biochemical processes, such as gene expression and translation, accumulation of compatible solutes, membrane lipid unsaturation, protein stability and antioxidant activity^10^. Plant noncoding RNAs play a significant role in the response to various abiotic and biotic stresses and are categorized into classes including microRNAs (miRNAs ~21–24 nucleotides in length), circular RNAs (circRNAs), long noncoding RNAs (lncRNAs) and small interfering RNAs (siRNAs)^11^, which use various molecular mechanisms to perform their important functions, ranging from modulation of RNA stability, translation, post-transcriptional and transcriptional regulation of gene expression^12,13^. Most of the identified miRNAs come from intergenic regions, but some conserved miRNAs have been derived from introns^14^. Several studies have reported various miRNAs involved in controlling various functions in Arabidopsis plants in response to heat stress^15^, miRNAs and their targets may play critical roles in stress resistance, including over-oxidation, phosphate starvation, polarity formation and morphogenesis, and hormone biosynthesis and signaling^16,17^. Recently, degradome analysis and high-throughput sequencing identified numerous miRNAs that might have essential functions in the heat-stress response of plants^18–20^. Using genomic and sequencing technologies, several miRNAs and their potential targets that play key roles in heat stress have been extensively studied in model plant species of various agricultural and vegetable crops^21^. Further studies have explored miRNA functions, including functional analysis of non-conserved and conserved miRNAs, their specific differential expressions and the quantification of the effects of miRNAs on their targets, which have highlighted their involvement in complex miRNA-mediated regulatory networks that play an essential role in controlling heat responses and tolerance^22–24^. Therefore, identification of the molecular mechanisms of heat tolerance of flowering Chinese cabbage is becoming an important research topic that will facilitate genetic improvement and production of this crop.

In our previous study, we reported express sequence tag-simple sequence repeat (EST-SSR) markers derived from RNA-seq of flowering Chinese cabbage after heat treatment. Those markers are valuable resources for genetic diversity analysis^1^. The identification of novel and conserved miRNAs that are responsive to heat stress offers an opportunity to better understand the molecular mechanisms of heat stress in flowering Chinese cabbage. To the best of our knowledge, no studies have identified heat-responsive miRNAs in flowering Chinese cabbage. This study aimed to identify novel miRNAs involved in heat stress, their differential expression, putative targets, and miRNA-related regulatory pathways linked to heat stress in flowering Chinese cabbage.

## Results

### Effect of heat stress on plant growth and antioxidant enzyme activity

**Figure 1:**
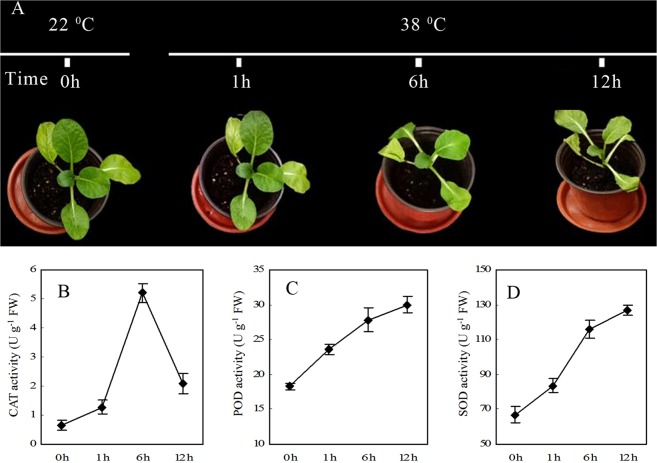
Physiological responses of flowering Chinese cabbage to heat stress.

High temperature had a marked impact on plant growth and development. Plant leaves showed wilting symptoms after exposure to high temperatures. The slight leave wilting was observed in heat treated group at 1 h of exposure to 38 °C, and then became very prominent at 6 h and 12 h (Fig. 1A), indicating that high temperature exereted severe water loss and inhibited growth of flowering Chinese cabbage. The activity of catalase (CAT) was significantly higher in leaves at 1 h, 6 h and 12 h than that in the 0 h control plants (Fig. 1B), with a maximum increase in CAT activity observed at 6 h of heat stress. In contrast, superoxide dismutase (SOD) and peroxidase (POD) activities in heat treated plants increased at 1 h, 6 h and 12 h compared to 0 h control plants (Fig. 1C,D). The findings showed that heat stress significantly increased CAT, SOD and POD activities in flowering Chinese cabbage.

### sRNA sequencing in response to heat stress

Sequencing four libraries of flowering Chinese cabbage genotype Youlv 501 collected at 0 h, 1 h, 6 h and 12 h after heat stress generated 28,194,816, 29,746,062, 28,002,965 and 29,049,979 raw reads, respectively. After removing reads with low-quality sequences—those without a 3′ primer or insert tag, shorter than 18 nt and 5′ contaminants—26,293,810 (93.26%), 27,726,336 (93.21%), 25,883,914 (92.43%) and 26,644,471 (91.72%) clean reads remained for tissue collected 0 h, 1 h, 6 h and 12 h after heat stress, respectively (Table 1).

**Table 1:** Numbers of sequence reads with varied quality in the small RNA databases of flowering Chinese cabbage from four libraries.

Sample	Total raw read count	Low-quality read count	Invalid adapter read count	Poly-A read count	Short valid length reads	Total clean read count	Percentage of clean reads (%)
0 h (control)	28,194,816	476,790	395,757	775	1,027,684	26,293,810	93.26
1 h	29,746,062	619,057	416,205	684	983,780	27,726,336	93.21
6 h	28,002,965	568,450	423,553	746	1,126,302	25,883,914	92.43
12 h	29,049,979	486,853	442,446	726	1,475,483	26,644,471	91.72

**Figure 2:**
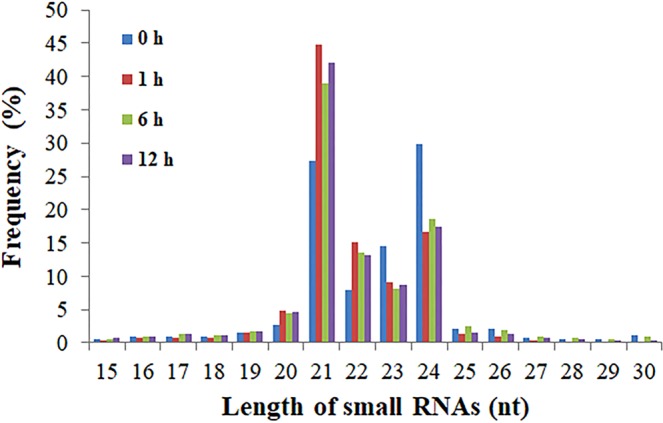
Sequence read length distribution of heat stress responsive small RNAs (sRNAs) in the libraries of flowering Chinese cabbage.

Subsequently, any sRNA reads that might belong to small nucleolar RNA (snoRNA), known rRNA, small nuclear RNA (snRNA), tRNA and unannotated reads were excluded (Table S1), leaving 20,775,702, 12,345,347, 12,704,323 and 14,705,931 mappable small RNA sequences in the 0 h, 1 h, 6 h and 12 h libraries, respectively, for further consideration. The sRNAs varied extensively in their length distribution in the four libraries, ranging from 15 to 30 nt (Fig. 2), with most ranging from 21–24 nt. Details of the sRNA length distributions with read counts in each library are in Table S2.

### Conserved miRNAs responsive to heat stress

**Figure 3:**
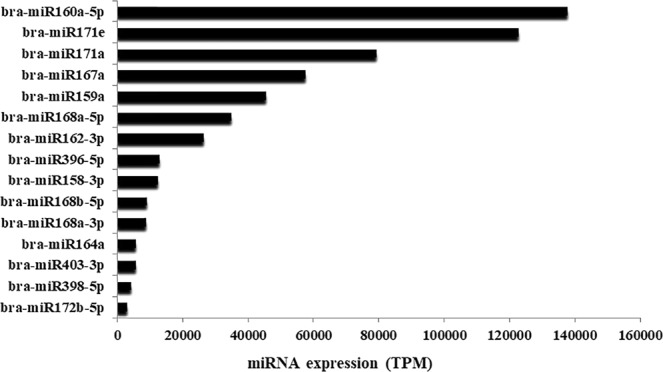
Top 15 abundantly expressed conserved miRNAs in flowering Chinese cabbage.

To examine conserved miRNAs in flowering Chinese cabbage, the sRNAs were exposed to a Blastn search against conserved miRNAs in miRBase 17.0, following no more than three mismatches. The length distribution of conserved miRNAs mostly occurred from 21–24 nt. To ensure the reliability of the results, conserved miRNAs with read counts <10 were excluded. The search identified 41 mature miRNA sequences of conserved miRNAs (details in Tables S3), of which bra-miR398-5p, bra-miR160a-5p, bra-miR168a-3p, bra-miR167a, bra-miR172b-5p, bra-miR159a, bra-miR162-3p, bra-miR403-3p, bra-miR396-5p, bra-miR158-3p, bra-miR168b-5p, bra-miR171a, bra-miR164a, bra-miR171e, and bra-miR168a-5p had very high expression levels (Fig. 3). Nineteen MIR families were identified from the sequencing analysis, of which MIR168, MIR156, MIR159, MIR171 and MIR1885 were the most abundant (Table S3).

### Novel miRNAs responsive to heat stress

To identify novel miRNAs involved in heat stress in flowering Chinese cabbage, small RNA mappable sequences were blasted against the *Brassica* database and then the miRBase to search for previously known miRNAs. The small RNAs not mapped as conserved miRNAs but exactly mapped to the genome sequence were considered as putative novel miRNAs. To enhance predictive accuracy in the identification of novel miRNAs, the miRNA/miRNA* criterion was the key consideration in our initial analysis. Using high-throughput sequencing, both candidate miRNA* and candidate miRNA sequences must be identified. miRNA and miRNA* must establish a duplex with nucleotides of 3′ overhangs in opposite stem-arms, mismatched bases must be <3, and the number of asymmetric bulges must be ≤1. Also, miRNA precursors must have minimal folding energy indices (MFEI) and higher negative minimal folding energy (MFE) values to differentiate from other small RNAs. Eighteen potential novel miRNAs were identified from the four libraries (Table 2).

**Table 2:** Sequences and chromosome locations of novel miRNAs from flowering Chinese cabbage under heat stress.

Name	Location	Length	Strand (+/−)	Mature sequence
Novel-mir001	A01:13241536–13241782	23	+	ATTTTGAGCATGTGGCTAGCTTT
Novel-mir002	A03:32323869–32324023	20	+	ATTCATGCTTGACTACATCA
Novel-mir003	A03:2196941–2197178	24	+	CGTAGACGCCGACGCAGTTTTAAT
Novel-mir004	A04:3008688–3008993	21	+	ACAACACTCCCACAAGACGAG
Novel-mir005	A04:3841793–3841922	21	+	ATGGCAGCCCATGTTAACATG
Novel-mir006	A05:11981503–11981733	22	−	CGGTGGAAGGATTACGGGCCAG
Novel-mir007	A05:4940093–4940268	23	+	CGTACCGCACCGCAGTTAACAGT
Novel-mir008	A05:662730–662965	24	+	GTATCTTGGCCCCACAGAAGTGAT
Novel-mir009	A06:15296650–15296806	23	+	TTGACGTAATCAGCCTCCAAATA
Novel-mir010	A06:12979559–12979722	23	−	CCTCAGACCTGGATGTAGAAGTT
Novel-mir011	A06:16377258–16377447	25	−	GCCCTATGAGATCCATTCCTGACGG
Novel-mir012	A08:9348257–9348369	25	+	CTTCTCCAAGGCCTCACTTATCACC
Novel-mir013	A09:31314690–31314867	22	+	TTTAACAGAGAGTAGAAACGGA
Novel-mir014	A09:24445902–24446047	24	−	GGCGTCAGCGTCAATGAAAATAGT
Novel-mir015	A09:9886482–9886659	23	+	TACCATACTGCCTAAGCCGAGTT
Novel-mir016	A09:33071308–33071419	22	+	TATTGGCCTGGTTCACTCAGAT
Novel-mir017	A09:8151167–8151329	24	+	GGTTTGAAGCGGTTTAGAACGATT
Novel-mir018	A10:19828692–19828861	24	+	TCCGCCCCTGGGTTCGAGCCTTGG

The mean MFE of predicted pre-miRNAs was −38.27 kcal/mol (range −19.7 to −207.4 kcal/mol). The MFEI value ranged from 0.8 to 2.2, with an average of 1.4, indicating reliable key characteristic features of the predicted miRNAs. The length distribution of most of the novel identified miRNAs ranged from 21–24 nt. Novel-mir003, novel-mir009 and novel-mir016 were most abundantly expressed in the 1 h, 6 h and 12 h libraries, compared to 0 h.

### Identification of differentially expressed miRNAs

**Figure 4:**
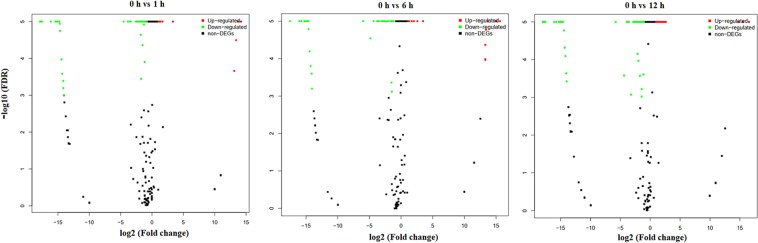
Volcano plots of differentially expressed heat-stress responsive miRNAs in flowering Chinese cabbage.

The conserved miRNA differential expression analysis revealed 16, 19 and 29 miRNAs with significant differential expression at 1 h, 6 h and 12 h, respectively, relative to 0 h (Fig. 4), of which 11, 16 and 24 were downregulated and 5, 3 and 5 were upregulated, respectively (Table S4). Higher differential expression values occurred in three upregulated miRNAs (bra-miR5725, bra-miR5726 and bra-miR172c-3p) and four downregulated miRNAs (bra-miR390-3p, bra-miR156a-3p, bra-miR158-5p and bra-miR9557-3p).

The novel miRNA differential expression analysis identified 15, 18 and 18 miRNAs with key differential expression at 1 h, 6 h and 12 h, respectively, relative to 0 h (Fig. 4), of which 12, 12 and 11 were downregulated and 3, 6 and 6 miRNAs were upregulated, respectively. In general, the number of downregulated miRNAs was higher at all time points (Table S5).

**Figure 5:**
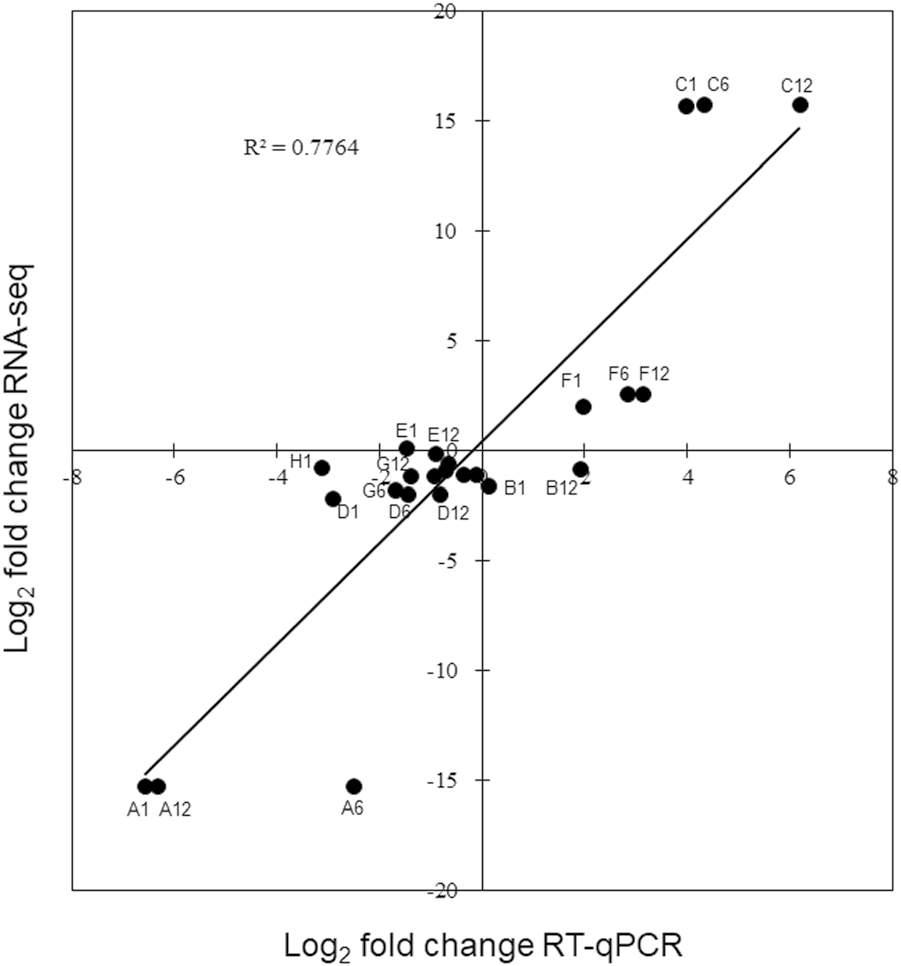
Validation of RNA-Seq results by RT-qPCR. Correlation between RNA-Seq and RT-qPCR data. The expression of eight miRNAs was validated using RT-qPCR. Letters A to H represent the miRNA names novel-mir001, novel-mir007, novel-mir013, novel-mir017, bra-miR160a-3p, bra-miR159a, bra-miR158-5p and bra-miR164a, respectively. Numbers 1, 6 and 12 represent the corresponding time-points of 1 h, 6 h and 12 h. The RT-qPCR log_2_-fold change values (X-axis) were plotted against RNA-seq data (Y-axis).

To validate the sequencing results, eight miRNAs (four novel and four conserved miRNAs), whose expression level covered both up- and down-regulated changes identified in the sequencing analysis, were randomly chosen for validation using RT-qPCR. The high correlation coefficient (R^2^ = 0.776) between RNA-seq and RT-qPCR expression profiles is shown in Fig. 5, indicates that trends in expression level for the selected miRNAs from RT-qPCR were consistent (up-regulation or down-regulation) with those obtained by sequencing data, and thus directly confirms that sequencing analysis effectively-identified differentially expressed miRNAs of flowering Chinese cabbage with appropriate sensitivity.

### Predicted target mRNA of the novel and conserved miRNAs

**Figure 6:**
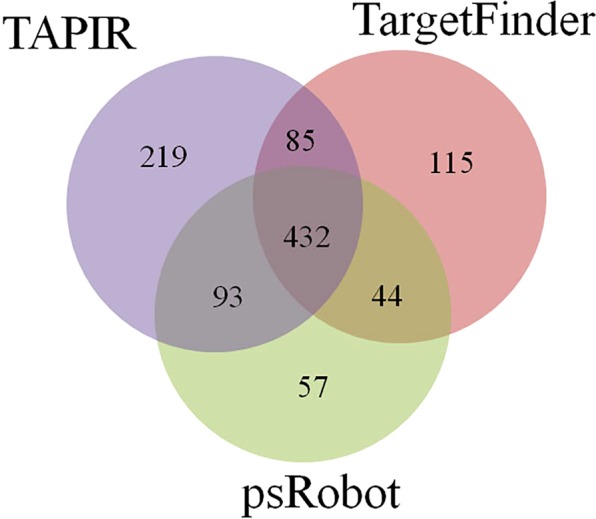
Comparison of numbers of predicted putative target mRNAs of heat-stress responsive miRNAs in the four flowering Chinese cabbage libraries among three prediction methods.

The identification of novel and conserved miRNA targets is essential for highlighting their potential functions in response to heat stress. We used TAPIR, TargetFinder and psRobot algorithms to screen and predict potential binding sites to annotate the mRNA targets for novel and conserved miRNAs response to heat stress. We identified 85 common spots between TAPIR and TargetFinder, 93 between TAPIR and psRobot, and 44 between TargetFinder and psRobot (Fig. 6), all of which were considered for further analysis.

### KEGG pathway analysis of target genes

**Figure 7:**
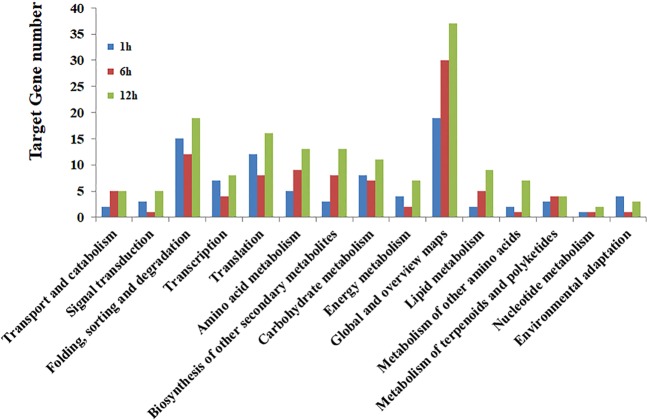
KEGG pathway analysis of target genes in heat-stress responsive miRNAs in the four flowering Chinese cabbage libraries.

To investigate the molecular interaction networks and biological and cellular functions of miRNA responsive target genes, we used KEGG pathway analysis. The miRNA target genes were differentiated into groups using KEGG pathway analysis. The key targets for cellular processes and environmental information processing were signal transduction, transport and catabolism (Fig. 7), and for genetic information processing were folding, translation, transcription, sorting and degradation.

In the global and overview maps, amino acid and carbohydrate metabolism are important gene repertoire from metabolism while environmental adaptation is a significant gene from organismal systems, respectively, in the 1 h, 6 h and 12 h treatment. Detailed information on the KEGG category gene repertoires in all three treatments are listed in Table S6.

**Figure 8:**
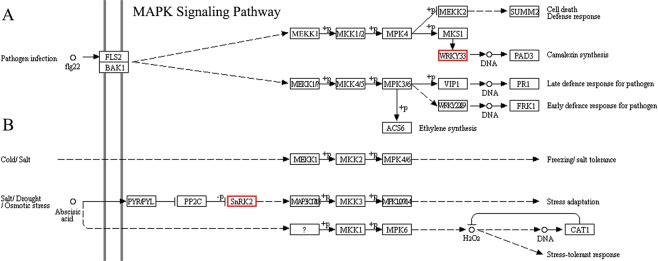
KEGG pathway map for MAPK signaling for flowering Chinese cabbage libraries. The KEGG pathway analysis also identified miRNA-regulated pathways from the predicted genes targeted^54^.

The KEGG pathway analysis also identified miRNA-regulated pathways from the predicted genes targeted and revealed that target genes were enriched for stress adaptation, stress tolerance, stress-tolerance response, and several MAPK signaling pathways, including cell death and early or late defense response for the pathogen (Fig. 8).

## Discussion

Flowering Chinese cabbage is a significant vegetable crop that is commonly cultivated in southern China due to its favorable taste and nutrient value^1^. Climate changes and global warming cause cellular homeostasisthat depresses plant growth and development and increases disease infection^25,26^. Numerous stress-regulated miRNAs have been identified and functionally characterized in many vegetable crops under different abiotic and biotic stress conditions such as heavy metal homeostasis, salinity, heat, nutrient deficiency, drought, UV-B radiation and bacterial infection^27–29^. To investigate the molecular mechanisms underlying plant growth and development after heat stress, high-throughput sequencing and bioinformatic approaches have been used to identify novel and conserved miRNA in model organisms and important food crops^30,31^. To the best of our knowledge, only 66 *B. campestris* miRNAs have been reported in the plant microRNA database^32^.

Yu *et al*.^33^ identified 21 novel miRNAs from 19 miRNA families, of which four were responsive to heat in *B. rapa*, particularly bra-miR5718 and bra-miR1885b.3. Furthermore, high-throughput sequencing of *B. rapa* identified 125 novel and 221 conserved miRNAs that play a significant role in stress responses, metabolism, and growth and development^34^. Using a comparative genomics-based approach, 126 novel miRNAs have been identified in *B. juncea* that play significant roles in various biological processes including high temperature, drought and salinity, of which miR156, miR160 and miR164 were predicted to target SPL2-like, ARF17-like and a NAC (No Apical Meristem) domain-containing proteins, respectively^35^. In the current study, we identified 18 novel and 41 conserved miRNAs responsive to heat stress in flowering Chinese cabbage, and the most abundant sRNAs were 21 to 24 nt long (Fig. 1), which is similar to those reported in *Brassica rapa*^33^ and *B. oleracea*^36^.

In our study, novel had less than 100 reads while most of the conserved miRNAs had more than 1000 reads. The highest numbers of reads were for bra-miR160a-5p (137,852 reads) and bra-miR171e (122,730 reads), which is similar to those detected in bra-miR159, which was almost near 45,000^32^. Furthermore, miR157a, miR166a, miR167a, miR168c and miR172a had the most sequencing reads in Brassicaceae species^37,38^. In the current study, we found 19 MIR families with MIR168, MIR156, MIR159, MIR171 and MIR1885 the most abundant families (Table S3). In terms of MIR families, MIR158, MIR166, MIR167, MIR169, MIR391, MIR838, MIR824, MIR1140 and MIR1885 are most abundant^11,27,38^. In a study on *B. oleracea*, miR157a, miR166, miR167 and miR168c were the most abundant molecules and MIR166, MIR167 and MIR169 the largest miRNA families^39^. Similarly, the comparative genome-based computational analysis identified 193 potential miRNAs from genome survey sequences and expressed sequence tags in *B. oleracea*. MIR158, MIR156, MIR169 and MIR5021 were identified as the largest miRNAs families that have various functions in the regulation of different stimuli^40^.

miRNAs exhibit fluctuating expression patterns over time during expression profiling. In the present study, 16, 19, and 29 conserved miRNAs and 15, 18, 18 novel miRNAs exhibited differential expression at 1 h, 6 h and 12 h, respectively, relative to 0 h (Tables S4 and S5). To verify the sequencing results, eight miRNAs—whose expression levels represent up- and down-regulated changes identified in the sequencing analysis—were randomly chosen for validation using RT-qPCR. The log_2_-fold change values and correlation coefficient (R^2^ = 0.776) showed that expression profiles were consistent (up-/down-regulation) in the sequencing and RT-qPCR data. Similar studies have also validated the expression of several conserved and novel stress-responsive miRNAs using RT-qPCR^35,36,41^. Likewise, Jiang and colleagues identified 18 miRNAs with differential expression for pollen development in male sterile lines of *B. campestris*, relative to the male fertile line, of which three were downregulated and 15 were upregulated^32^. In recent years, numerous bioinformatics algorithms have been widely used for predicting plant miRNA targets, including TAPIR^42^, Target-align^43^ and psRNATarget^44^. In our study, the TAPIR, TargetFinder and psRobot algorithms identified 432 potential mRNA targets for novel and conserved miRNAs in *B. campestris*. Identification of target genes is the important and initial step to investigate the key function of miRNAs. Likewise, a computational approach and deep sequencing analysis identified 20 conserved miRNAs that exhibited significant differential expression between heat-sensitive and heat-tolerant genotypes under heat stress in *B. oleracea*^39^. Recently, different components in thermotolerance were identified that play key roles in the defense of oxidative damage^36^. The study revealed that the predicted miRNAs have an essential function in the regulation of target genes for shoot apical development, phase change, and hormone and energy metabolism, and the response to temperature stimulus^36^. Another study on *B. oleracea* predicted that miRNAs might target mRNAs encoding several potential proteins involved in the regulation of various functions, including abiotic stress response, oxidative phosphorylation, cell communication and hormone stimuli^39^. Several miRNAs have been identified as involved in lipid and fatty acid metabolism, including miR1134, miR156e-p3, miR838-p3, miR9563a-p3, miR159c, and miR9563b-p5 that could target WSD1, PlCD6, PDP, LACS9, ADSL, C0401, MFPA, OLEO3, ACO32 and GDL73 in *Brassica napus*^45^. Furthermore, Wang *et al*. (2012b) identified several miRNAs targets that participate in various functions, including plant development, signal transduction and stress response.

In conclusion, we have presented the first comprehensive heat-responsive small RNA dataset for *B. campestris* L. ssp. *chinensis* var. *utilis* Tsen et Lee. Using next-generation sequencing and a biocomputational approach, we identified 18 novel and 41 conserved miRNAs. The putative target prediction determined the roles and functions of the identified miRNAs in flowering Chinese cabbage. The identified miRNA sequences, their differential expression, and predictive mRNA targets presented in the current study can be used to plan crop development approaches in flowering Chinese cabbage and related species. Since the full genome of flowering Chinese cabbage had not yet been reported, the entire set of miRNAs and its possible targets could not be identified. However, this study has delivered significant information on the stated molecules and their involvement in different functions.

## Materials and Methods

### Plant materials, sample collection and total RNA extraction

Flowering Chinese cabbage (*Brassica campestris* L. ssp. *chinensis* var. *utilis* Tsen et Lee) genotype Youlv 501 was grown in a growth chamber at the experimental station of Guangzhou University set at 28/22 °C for 14/10 h (day/night). Plants were moved into another growth chamber set at 38/29 °C (14/10 h) at the five-leaf stage for the heat-stress treatment. Samples were collected from the upper fully expanded leaves at four time-points (0 h (control), 1 h, 6 h and 12 h after the start of the heat-stress treatment), flash-frozen in liquid nitrogen, and kept at −80 °C until further needed. Trizol RNA extraction kit (Invitrogen, USA) was used to isolate total RNA following the manufacturer’s instructions.

### Determination of antioxidant enzyme activities

The activity of antioxidant enzymes (SOD, POD and CAT) were measured using assay kits (Nanjing Jiancheng Bioengineering Institute, China) following the manufacturers’ instructions. Briefly, fresh leaves (0.5 g) were crushed with pestle and mortar and homogenized with 50 mM phosphate buffer (pH 7.0). The homogenate samples were centrifuged at 3,500 *g* for 10 min (4 °C). Enzyme activities were determined with a spectrophotometer using the supernatants. All procedures were carried out at 0–4 °C.

### Construction of sRNA libraries and sequencing

The RNA for each time point was isolated from five individual replicates and mixed in an equivalent proportion. The cDNA library was constructed using an Illumina TruSeq Small RNA Preparation Kit following the manufacturer’s recommendations. In brief, (1) RNA 3′- and RNA 5′-adapters were ligated to total RNA, (2) cDNA constructs were generated using reverse transcription after PCR, and (3) small cDNA fragments were isolated in different lengths (18–30 nt) using 6% denaturing polyacrylamide gel electrophoresis. The purified cDNA library was subjected to next-generation sequencing using Illumina HiSeq technology at the Beijing Genomics Institute (BGI, Shenzhen, China) following the manufacturer’s instruction for running the instrument. Raw sequencing was collected using Illumina’s analysis software.

### Bioinformatics analysis of sequencing data to identify novel and conserved miRNAs

Raw sequence reads were collected and analyzed after removing adapter sequences, oversized insertion tags, no-insert tags, 5′-primer contaminants, low-quality tags, poly-A tags and small tags (those without 3′ primers and sequences beyond 15–30 nucleotides). To analyze small RNA length distributions, the Bowtie2 web program was used to map clean reads to the reference genome and other sRNA databases^46^. The unique sRNAs were aligned to known non-codingRNAs downloaded from the Rfam database http://www.sanger.ac.uk/science/tools/rfam/ with use of the NCBI BLASTN to discard tRNA, rRNA, snRNA, scRNA and snoRNA. Matched sequences were excluded for further analysis, unmatched sequences were subjected to evaluation of matched and mismatched sequences through the *Brassica* database (http://brassicadb.org/brad/). The miRNAs were considered potential conserved candidates if they had no more than three nucleotide of mismatches, while candidate novel miRNAs were reserved in unmatched sequences. Finally, miRBase software was used to predict novel miRNAs from unannotated clean sequences^47^.

### Identification of differentially expressed miRNAs

The control and heat-stress treatments were compared to identify differential expression of miRNAs in flowering Chinese cabbage under heat stress. The levels of miRNA expression were normalized to transcripts per million in all libraries and converted to 0 to 0.01 to avoid calculation error. If the miRNA had normalized expression of <1 in all libraries, then it was excluded from further comparative analysis due to low expression. The normalization equation is:

Normalized expression = actual miRNA count/total count of clean reads × 10^6^.

The *P*-values and fold-change values were examined using normalized data, and the fold-change values used to generate a scatter plot:

Fold-change = log_2_ (treatment/control).

The *P*-value was determined as follows:

$$p(x|y)={(\frac{{N}_{2}}{{N}_{1}})}^{y}\frac{(x+y)!}{x!y!\,{(1+\frac{{N}_{2}}{{N}_{1}})}^{(x+y+1)}}$$

$$C(y\le \,{y}_{min}|{\rm{x}})=\mathop{\sum }\limits_{y=0}^{y\le {y}_{min}}p(y|{\rm{x}})$$

$$D(y\ge {y}_{max}|{\rm{x}})=\mathop{\sum }\limits_{y\ge {y}_{max}}^{\infty }p(y|x)$$

where *x* and *y* denote values of sRNA total clean reads in the control and treatment, respectively, and *N*_1_ and *N*_2_ represent miRNA normalized expression in the library control and treatment, respectively. The Bonferroni method was used to correct *P*-value parallels to differential gene expression^48^.

### Prediction of novel miRNAs targets in *Brassica campestris*

Three software packages were used to analyze and predict mRNA targets of differentially expressed miRNAs including TargetFinder^49^, TAPIR^42^, and psRobot^50^ as described earlier^51^. To achieve a more reliable result and to increase the confidence interval, only binding sites that were predicted by all three software packages were selected.

### KEGG prediction of miRNA-related regulatory pathways

Kyoto Encyclopedia of Genes and Genomes (KEGG) pathway (http://www.genome.jp/kegg/pathway.html) database was used to identify the microRNA-related regulatory pathways using a corrected *P*-value (≤0.05) with a threshold derived from a hypergeometric test.

### Real-time qPCR validation

To confirm the sequencing results, eight miRNAs, whose expression levels represent both up- and down-regulated changes identified in sequencing analysis, were randomly chosen for validation using RT-qPCR as described^52^. The RNA used for RT-qPCR were aliquots of RNA samples used for sequencing and included 0 h, 1 h, 6 h and 12 h RNA of flowering Chinese cabbage. Briefly, 1 µg of total RNA was reverse transcribed into cDNA with miRcute miRNA first-strand cDNA synthesis kit (TIANGEN Biotech, Beijing, China). Specific primers were used for qRT-PCR (Table S7). RT-qPCR was performed using LightCyclerR 480 SYBR Green I Master (Roche) following the manufacturer’s instructions. U6 served as an internal control. All reactions were carried out using two biological samples with three technological replicates, and novel and conserved miRNAs relative expression level at each time point was calculated using the 2^−ΔΔCT^ method^53^.

### Statistical analysis

The statistical analysis used SPSS software (version 22.0; IBM Corp., Armonk, NY, USA) and Student’s *t*-test difference between treatments. One-way analysis of variance used Tukey’s test for multiple comparisons at the *P* < 0.05 or *P* < 0.01 significance level. Data were expressed as means ± SEM.

## Supplementary information

Supplementary informartion
